# Randomized Controlled Trial of the Picture Book Reading Program on Cognitive Function in Middle-Aged People

**DOI:** 10.3389/fpsyt.2021.624487

**Published:** 2021-05-19

**Authors:** Ai Iizuka, Hiroyuki Suzuki, Susumu Ogawa, Tomoya Takahashi, Daisuke Cho, Daichi Yamashiro, Kenichiro Sato, Yan Li, Yuri Kanabe, Momoko Kobayashi, Yoshinori Fujiwara

**Affiliations:** Research Team for Social Participation and Community Health, Tokyo Metropolitan Institute of Gerontology, Tokyo, Japan

**Keywords:** cognitive function, cognitive intervention, middle-aged people, verbal fluency, picture book reading, randomized controlled trial

## Abstract

**Background:** To delay cognitive decline, it is important to engage actively in preventive activities from middle age (50–64 years of age). We have developed a cognitive intervention program using picture book reading, and demonstrated that it is effective for improving memory in older adults. However, the effect of the intervention on memory and other cognitive functions in middle-aged people has not been examined. The current study investigated the effects of the picture book reading program on cognitive function in middle-aged people.

**Methods:** This single-blind (examiners were blinded) randomized controlled trial was conducted in Tokyo, Japan. A total of 65 participants were randomly assigned to the intervention group (IG) (*n* = 32), in which members attended 12 picture book reading classes held once a week, or the active control group (CG) (*n* = 33), in which members received lectures on health maintenance. Cognitive tests were conducted before and after the intervention. The primary outcome was memory, and the secondary outcomes were verbal function and executive function.

**Results:** The results showed that there was no significant difference between the IG and the CG in change scores (post minus pre) for memory. On the other hand, there was a significant difference in change scores of the category fluency, which is a measure of verbal function, suggesting improvements in IG compared to CG. There were also no significant differences in executive function.

**Conclusions:** The results indicated that our previous finding of an improvement in memory function in older adults was not found in middle-aged people. However, the findings suggest that the picture book reading program may affect lexical access ability in verbal function among middle-aged people. Because maintaining verbal function is important for daily communication, these findings suggest that this program may be a useful countermeasure for cognitive decline in middle-aged people.

**Clinical Trial Registration:** University Hospital Medical Information Network Clinical Trial Registry, https://upload.umin.ac.jp/cgi-open-bin/ctr_e/ctr_view.cgi?recptno=R000048012, Identifier: UMIN 000042071

## Introduction

Aging is one of the major risk factor of cognitive decline (1). The population of people with dementia is rapidly increasing, constituting a major cause of long-term care and imposing a heavy economic and social burden (2, 3). Therefore, it is necessary to take measures against cognitive decline in both normal aging and dementia at an early stage (4).

Cognitive decline progresses slowly throughout life. There are various risk factors for cognitive decline, and several factors that can be modified (education, hypertension, diabetes mellitus, depression, and hearing loss) have recently been identified (1). To delay cognitive decline, it is important to actively engage in preventive activities from middle age (50–64 years of age) (5). Hence, the targets of measurement of cognitive decline have expanded to include middle-aged people in addition to older adults.

Non-pharmacological interventions that promote behavioral changes provide one approach for delaying cognitive decline (6). In addition to exercise and nutrition, interventions that include elements of intellectual activity and social interaction have attracted substantial research attention (4, 7–12). It has been reported that intellectual activity delays cognitive decline by strengthening synaptic transmission (i.e., neural plasticity) and increasing cognitive reserve (13, 14), and that frequent engagement in intellectual activity maintains cognitive function (15–17). Although there are many types of intervention involving intellectual activity, interventions that include elements of learning new skills and social interaction are considered to be particularly effective for cognitive function (18).

Based on this background, we developed a “picture book reading program,” an intellectual activity that includes elements of learning new skills and social interaction, and conducted an intervention study (19). The picture book reading program is a training program focused on acquiring the skills needed for reading picture books to children. To successfully read picture books to children, it is necessary to select a suitable picture book for the situation from a large number of books, to memorize the outline of the story, and to plan how to speak and show the pictures. The training program was designed to involve a high degree of intellectual stimulation.

A previous randomized controlled trial examining the effects of the picture book reading program on cognitive function revealed that the picture book reading program improved verbal memory in older adults aged 65 and over who were worried about forgetfulness, and that had a positive influence on executive function in people with mild cognitive impairment (19). These findings indicated that memorizing the contents of picture books had a positive effect on verbal memory and that reading picture books over time may have improved executive function. However, the effect of this program on cognitive function in middle-aged people (50–64 years of age), who have been the target of dementia prevention in recent years (5), has not been examined. Because picture book reading involves high-level cognitive activity, it may have a positive effect on verbal memory in middle-aged people, as well as older adults with normal cognitive function. However, the impact of picture book reading on verbal memory in middle-aged people is currently unclear, and its effect on other types of cognitive function remains to be elucidated. It is also important to examine the effectiveness of such programs on cognitive function in a way that can be applied in the real world.

Therefore, the purpose of the current study was to examine the effects of a picture book reading program on cognitive function in middle-aged people between 50 and 64 years old.

## Materials and Methods

### Study Design Participants

This single-blind (examiners were blinded) randomized controlled trial was conducted in collaboration with local governments in Tokyo, Japan. Interventions and outcome assessments were divided into five small groups between 2015 and 2019, with 15–20 participants in each group. We recruited participants in each group by publishing advertisements in community newspapers and in community letters published by the local governments. Because this study was part of a project conducted by the local government, we recruited participants without setting an upper age limit. Therefore, this intervention was originally planned for older adults, and the clinical trial registration includes those aged 65 and over. However, since the main purpose of this study is to evaluate the intervention effect on middle-aged people, we only included middle-aged people in the analysis presented in this paper.

The inclusion criteria were as follows: age 50–64 years old, being able to perform activities of daily living independently, and not having any cognitive impairment. The exclusion criteria were as follows: having medical and psychiatric disorders affecting cognitive function, having visual or hearing loss affecting daily life, and having a diagnosis of dementia.

The 65 participants, who obtained informed consent and met the inclusion criteria, were randomly assigned to one of the following two groups: (1) an intervention group (IG) (*n* = 32); (2) an active control group (CG) (*n* = 33). The random number generation method with the RAND function in Microsoft Excel 2010 and 2013 was used for randomization, and the order of application for the participants was concealed from the staff. The randomization, group allocation, and contacting of participants were conducted by local government staff, and the researchers who evaluated and analyzed the data were not involved in these processes. The examiners of the cognitive tests were blinded to the patient's status, but the participants and researchers could not be blinded. This study did not conduct a mid-intervention analysis because the intervention period was short (3 months) and there were few adverse events due to the intervention. The cancellation criteria were if the participants requested to withdraw from the study.

**Figure 1:**
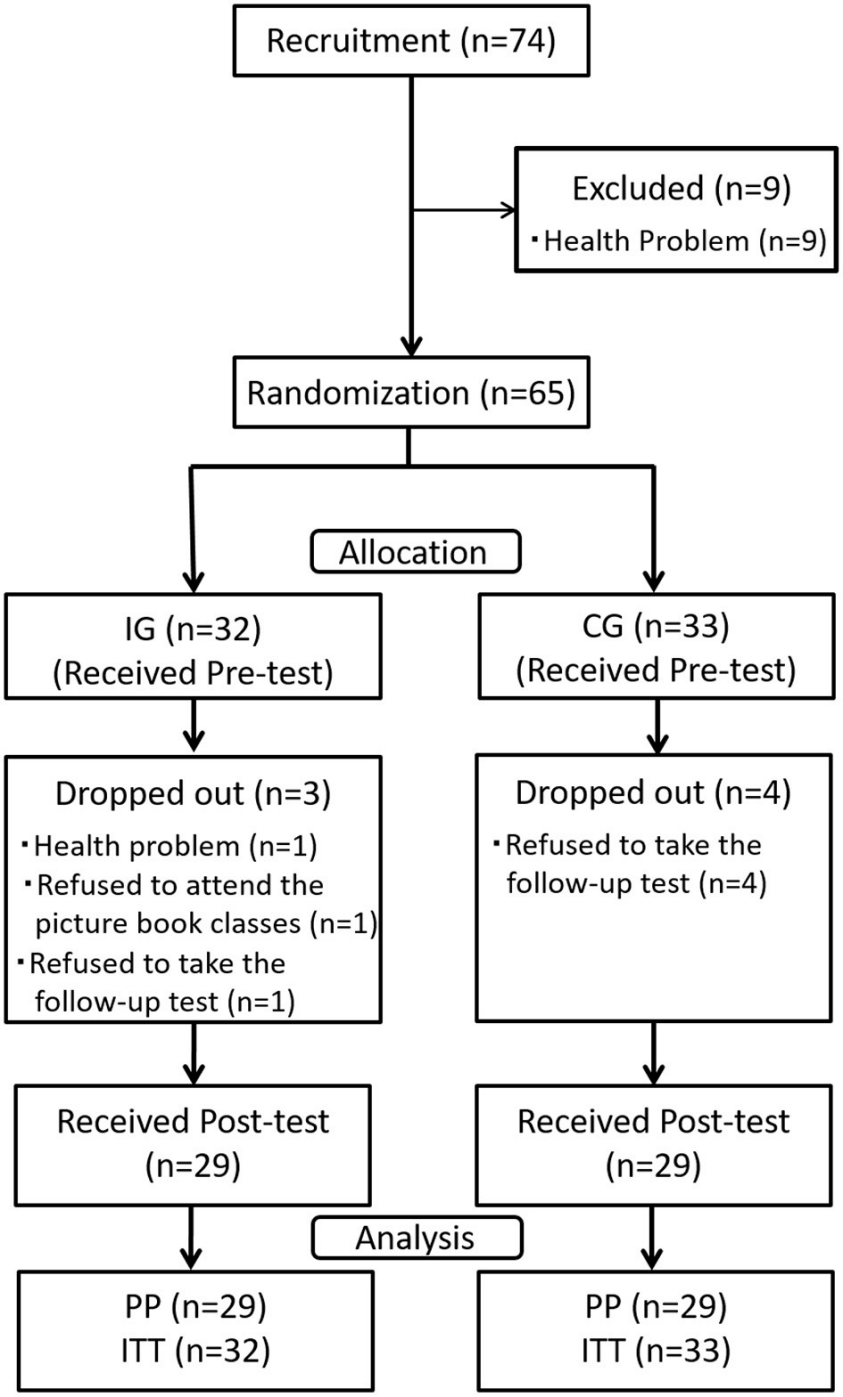
Consolidated standards of reporting trials (CONSORT) flow diagram of the study process. *N*, number of participants; ITT, intent-to-treat analysis; IG, intervention group; CG, active control group; PP, Per-protocol analysis.

This study was registered in the University Hospital Medical Information Network Clinical Trial Registry (UMIN). The trial design based on the Consolidated Standards of Reporting Trials (CONSORT) (20) is shown in Figure 1.

### Ethical Approval

Ethical approval was obtained from the Institutional Review Board and Ethics Committee of the TMIG (Acceptance No. 0083). We explained the purpose, methods, and ethical considerations of this study and obtained written informed consent from each participant based on the Declaration of Helsinki before enrolment.

### Interventions

#### Intervention Group

Participants attended twelve 2-h classes focused on learning and mastering methods of picture book reading, which were held once a week at a community center. The classes were run by an instructor skilled at picture book reading, and research staff. Specifically, the classes were designed to teach participants how to choose a book, how to memorize a story, how to show the pictures, how to enunciate the words, and how to read with feeling. The first half of the classes comprised individual learning and the second half comprised group learning in which participants discussed and collaborated with each other. In the final class, participants read several picture books in front of other participants, imagining that they were reading to a group of children.

The picture book reading program and the intervention method was the same as that used in our previous study for community-dwelling older adults (19). The program was based on the pilot study REPRINTS (Research of Productivity by Intergenerational Sympathy), which engages senior volunteers in picture book reading to children (21).

#### Active Control Group

Participants received a 2-h lecture on health maintenance, which was unrelated to picture book reading, covering topics such as exercise, nutrition, mental health, and social participation once a month. All participants took part in a total of three lectures. The only purpose of the lectures was to maintain motivation to participate in this study. This group is described as “placebo/sham control” in our clinical trial registration.

#### Measures

We set outcome measures for each cognitive domain. The main outcome was memory function, which was found to be impacted by the program in our previous study of older adults (19), and the secondary outcomes were verbal function and executive function. We conducted cognitive tests before and after the intervention period (3 months). Cognitive tests were conducted by testers who were blinded to the participant groups.

#### Baseline Characteristics

We obtained participants’ baseline characteristics, including age, sex, education level, past medical history, medication history, and mental health, using the 15-item Geriatric Depression Scale (GDS-15) (22). We assessed global cognition as a baseline characteristic using the Mini-Mental State Examination-Japanese (MMSE) (23, 24), which is a screening tool for dementia. The maximum score was 30 points, and the cutoff score was 23/24.

### Assessment of Cognitive Function

#### Memory

The logical memory (LM) I and II subscales of the Wechsler Memory Scale-Revised were used to assess immediate and delayed memory (25). Participants were asked to remember the content of two short stories. In LM I, the examiner read the stories aloud, and the participant was required to repeat both stories immediately. In LM II, the participant was asked to recall both stories 30 minutes later. The maximum score for both tests was 50, with higher scores indicating better memory. In addition, we calculated ΔLM as memory retention rate by dividing LM II by LM I.

#### Verbal Function

Verbal fluency tests (26–28) were used to assess verbal function. The participants were asked to generate as many words as possible, starting with a specific Japanese hiragana syllable (a, ka, ho) (Letter fluency) or belonging to the same category (“animals” and “vegetables”) (Category fluency) within 1 min. In letter fluency, two out of three syllables, “a,” “ka,” and “ho” were examined for each participant, and the total scores of the two syllables were used for the analysis. It was used as an indicator of language executive control ability (27). In category fluency, the total scores of animals and vegetables were used for the analysis. This task was used as an indicator of lexical access ability, and it resembles an everyday word production task (27). Higher scores indicate better verbal function.

#### Executive Function

The Trail Making Test Part A and B (TMT-A and TMT-B) (28, 29) were used to assess executive function. TMT-A and B comprise 25 scattered circles written on the examination paper. In TMT-A, the circles included numbers from 1 to 25, and the participant was asked to draw lines to connect the numbers in order as quickly and accurately as possible. It was used as an indicator of processing speed. In TMT-B, the circles included either numbers 1 to 13 or the first 12 letters of the Japanese hiragana syllables. Participants were asked to connect the numbers and hiragana letters alternately (for example, participants connected “1” with “a,” “2” with “i”) as quickly and accurately as possible. It was used as an indicator of working memory. Faster performance indicated higher executive function.

#### Sample Size

Our sample size estimation is based on the change score in the LM, which is the primary outcome in this study. We expected to detect a medium effect size (*f* = 0.29) of the change score in the LM between the IG and the CG based on our previous study for older adults and other cognitive intervention studies. The sample size was determined using G ^*^ power based on 80% power, a two-sided hypothesis test, an alpha level of 5%, and a one-way analysis of covariance (ANCOVA) model. Based on the effect size, detection power, and significance level, the rejection limit was calculated as 3.94, the non-centrality parameter λ was 8.07, and the degree of freedom of the residual was 90. The sample size calculation indicated that we need 128 participants, with consideration of a 25% drop-out rate, but in this paper, the sample size is smaller than that listed in the original research plan because only middle-aged people were included for this analysis.

### Statistical Analysis

#### Cognitive Function

To assess the effects of the intervention on cognitive function, we calculated change scores (post-test scores minus pre-test scores), and conducted an analysis of covariance (ANCOVA) for each cognitive test. We also performed a lack of fit test for each model as a prerequisite for performing ANCOVA. The change scores were set as the dependent variable. Group (IG and CG) was set as the independent variable. To adjust for the background characteristics, we analyzed model 1, in which the pre-test scores are set as covariates, and model 2, which adjusts for sex, age, and educational level along with the pre-test scores, which generally affect cognitive function. Further, we calculated partial eta squared (ηp^2^) as an index of effect size (30).

We performed a per-protocol analysis (PP) including the participants who attended the intervention with an attendance rate of 70% or higher and completed the post-test. Multiple imputations were implemented with a model that combined the IG and CG (rather than use separate models for each group) assuming that unobserved data were randomly missing and post-test data were entered regardless of why they were missing. After multiple imputations, an Intent-to-Treat (ITT) analysis was also performed on the two models; Model 1 was adjusted by the pre-test and Model 2 was adjusted by sex, age, and educational level along with the pre-test scores, similar to the PP analysis. For multiple imputations, 100 data sets were generated with the imputation of missing values using predictive mean matching via the “mice” library in R (R Development Core Team, Vienna, Austria) (31, 32).

Since each cognitive test measures different aspects of cognitive function, and there is no overlap in specific cognitive area, we considered it was not necessary to adjust the multiplicity. Therefore, we regarded a two-sided *p* < 0.05 as statistically significant. Statistical analyzes were conducted using SPSS version 23 (IBM Inc., Chicago, IL), and R.

## Results

### Compliance With the Program

Three participants in the IG and four in the CG dropped out of the intervention program for the following reasons: (1) in the IG, one participant was hospitalized with a severe medical disorder, one refused to attend the picture book reading classes, and one refused to participate in follow-up assessment; and (2) in the CG, four participants refused to participate in the follow-up assessment. Therefore, 29 participants in IG and CG, respectively, were included in the PP analysis. The overall attendance rate of the picture book reading classes was 96.5%, and none of the participants had attendance rate below 70%. The participant characteristics of those who completed the post-test are described in Table 1, and those who dropped out are described in Supplementary A.

**Table 1:** Baseline characteristics in both intervention and control groups.

		**IG (*n* = 29)**	**CG (*n* = 29)**
Age (mean ± SD)	Years	59.9 ± 4.3	60.6 ± 3.2
Sex (male/female)	*N*	1/28	1/28
Education (12/13 years)	*N*	4/25	7/22
GDS-15 [≦4/5≦ (median)]	*N* [Score (0–15)]	23/6 (3.0)	23/6 (3.0)
MMSE [≦23/24≦ (median)]	*N* [Score (0–30)]	29/0 (29.0)	29/0 (30.0)

*N, number of participants; SD, standard deviation; IG, intervention group; CG, active control group; GDS-15, 15- item geriatric depression scale; MMSE, mini-mental state examination*.

### Effects on Cognitive Function

The pre-intervention and post-intervention scores in cognitive functions are presented in Table 2. An independent samples *t*-test was performed to compare the pre-test scores of IG and CG, and no significant differences were found between the two groups.

**Table 2:** Cognitive test scores of participants who completed the pre-test and post-test.

		**IG (*****n*** **=** **29)**		**CG (*****n*** **=** **29)**		***p*-value**^**b**^
		**Pre**	**Post**	**Pre**	**Post**	
**Memory**						
LMI	Score (0–50)	22.5 ± 6.7	25.9 ± 5.2	22.3 ± 5.6	26.9 ± 6.3	0.91
LMII	Score (0–50)	17.0 ± 7.3	22.3 ± 7.3	17.6 ± 6.5	22.7 ± 6.3	0.70
ΔLM	%	0.7 ± 0.2	0.8 ± 0.2	0.7 ± 0.1	0.8 ± 0.1	0.40
**Verbal function**						
Category	Number of words	38.8 ± 8.9	41.5 ± 8.5	37.6 ± 5.8	37.5 ± 5.1	0.53
Letter	Number of words	24.9 ± 6.1	26.9 ± 6.8	24.6 ± 8.9	26.4 ± 7.6	0.87
**Executive function**						
TMT-A^a^	Seconds to completion	34.0 ± 10.0	30.2 ± 6.6	34.1 ± 10.9	31.0 ± 9.9	0.97
TMT-B^a^	Seconds to completion	71.0 ± 20.2	72.4 ± 21.3	77.2 ± 28.1	67.9 ± 16.9	0.35

*N, number of participants; SD, standard deviation; IG, intervention group; CG, active control group; LM, logical memory; Category, category fluency; Letter, letter fluency; TMT, trail making test*.

a *Since two participants in the IG could not be evaluated accurately TMT-A and TMT-B, the number of participants of IG is 27*.

b *p-values are from independent sample t-tests*.

^*^p < 0.05.

To detect intervention effects on cognitive function, we conducted ANCOVA on the change scores in each cognitive test (Table 3).

**Table 3:** Cognitive test change scores of participants who completed the pre-test and post-test.

		**IG**		**CG**		**Model 1**			**Model 2**		
		**Mean ± SD**	**95%CI**	**Mean ± SD**	**95%CI**	***F*-value**	***p*-value**	**Effect** **size** **(*ηp***^**2**^**)**	***F*-value**	***p*-value**	**Effect** **size** **(*ηp***^**2**^**)**
**Memory**											
LM I	Score	3.4 ± 5.2	1.4–5.4	4.5 ± 4.7	2.7–6.4	0.83	0.36	0.01	0.67	0.41	0.01
LMII	Score	5.3 ± 3.0	4.1–6.4	5.1 ± 5.8	2.8–7.3	0.00	0.97	0.00	0.03	0.86	0.00
ΔLM	%	0.1 ± 0.1	0.0–0.1	0.0 ± 0.2	−0.0 to 0.1	0.19	0.65	0.00	0.32	0.57	0.00
**Verbal function**											
Category	Number	2.6 ± 7.8	−0.3 to 5.6	−0.1 ± 3.9	−1.5 to 1.3	5.21	0.02^*^	0.08	4.55	0.03^*^	0.08
Letter	Number	2.0 ± 5.1	0.0–3.9	1.8 ± 7.4	−1.0 to 4.6	0.05	0.82	0.00	0.01	0.91	0.00
**Executive function**											
TMT-A^a^	Seconds	−3.8 ± 7.3	−6.7 to 0.8	−3.1 ± 9.8	−6.8 to 0.6	0.15	0.69	0.00	0.02	0.87	0.00
TMT-B^a^	Seconds	1.4 ± 23.6	−7.8 to 10.8	−9.2 ± 24.3	−18.4 to 0.0	1.91	0.17	0.03	2.49	0.12	0.04

*SD, standard deviation; IG, intervention group; CG, active control group; Category, category fluency; CI, confidence interval; Letter, letter fluency; LM. logical memory; TMT, trail making test*.

a *Since two participants in the IG could not be evaluated accurately TMT-A and TMT-B, the number of participants of IG is 27*.

*Model 1: The results of analysis of covariance, adjusted for pre-test scores*.

*Model 2: The results of analysis of covariance, adjusted for sex, age, and educational level along with the pre-test scores*.

* *p < 0.05*.

The PP analysis for memory, which was the main outcome of this study, there was no significant difference between the IG and the CG with LM I, LM II, and ΔLM [respectively, Model 1: *F*_(1, 52)_ = 0.83, *p* = 0.36; *F*_(1, 52)_ = 0.00, *p* = 0.97; *F*_(1, 52)_ = 0.19, *p* = 0.65; Model 2: *F*_(1, 52)_ = 0.67, *p* = 0.41; *F*_(1, 52)_ = 0.03, *p* = 0.86; *F*_(1, 52)_ = 0.32, *p* = 0.57].

In verbal fluency, the results showed a significant difference between the groups in the change scores for category fluency (regarded as a measure of lexical access ability), and scores in the IG improved compared with those in the CG [Model 1: *F*_(1, 52)_ = 5.21, *p* = 0.02; Model 2: *F*_(1, 52)_ = 4.55, *p* = 0.03]. On the other hand, there was no significant between-group difference in score changes of letter fluency [Model 1: *F*_(1, 52)_ = 0.05, *p* = 0.82; Model 2: *F*_(1, 52)_ = 0.01, *p* = 0.91].

In executive function, there were also no significant differences in score changes between groups with TMT-A and TMT-B [respectively, Model 1: *F*_(1, 52)_ = 0.15, *p* = 0.69; *F*_(1, 52)_ = 1.91, *p* = 0.17; Model 2: *F*_(1, 52)_ = 0.02, *p* = 0.87; *F*_(1, 52)_ = 2.49, *p* = 0.12].

We additionally performed ITT analysis by complementing the missing values by the multiple imputation method, and it showed almost the same results as PP analysis in all outcomes (Supplementary B).

## Discussion

The purpose of the current study was to examine the effects of a picture book reading program on cognitive function in middle-aged people. The results indicate that our previous finding of improved memory function in older adults who were worried about forgetfulness was not found in middle-aged people in both the PP and ITT analysis. The results suggest that the picture book reading program may affect lexical access ability in verbal function among middle-aged individuals. Although people accumulate a substantial amount of word knowledge throughout their lives, it is widely accepted that the ability to search and generate words decreases with age (33, 34). Therefore, maintaining verbal function is important for continuing smooth communication.

In our previous study, the picture book reading program was found to improve memory in older adults who were worried about forgetfulness (19). Thus, we hypothesized that the program would also improve memory in middle-aged people. However, the current study revealed no significant differences in memory function changes between the IG and the CG. A measure of LM that is commonly used as an outcome of memory is a task in which short stories are memorized, and a learning effect is often observed by repeating the test in a short period of time (35, 36). It is not certain because the effect over time (the effect within the group) has not been assessed, but it is possible that such a learning effect appeared, and influenced the change scores. Because the participants were young and had relatively high levels of cognitive functioning, they had less intervention effect on memory from the program than older people and might have easily obtained learning effects of LM.

In the program examined in the current study, participants were exposed to many words through reading picture books aloud many times. Participants in the IG appeared to be able to recall many words by repeating these tasks, improving lexical access, expanding the scope of vocabulary search, and making the words available in an efficient way. A previous study reported that training in an intervention involving repeated reading of poetry improved verbal fluency (37), and another previous study suggested that there is a possibility of an association between verbal fluency and reading books frequently and reading letters repeatedly (38). The results of these previous studies support the finding of the present study. Furthermore, the results of the picture book reading program may have also been affected by the increased opportunities for communication by talking with other participants during the group activities.

The current results revealed no significant differences in letter fluency. Letter fluency requires language executive control ability and flexibility in vocabulary search, such as setting and switching the rules of searching words (26, 27). Because the picture book reading program involves training to read a fixed sentence repeatedly, the flexibility of vocabulary search, involving switching tasks quickly, may not have been strongly influenced by the intervention.

Whereas, our previous study showed that the picture book reading program improved executive function in participants with mild cognitive impairment (19), the current study did not show any intervention effect on executive function in middle-aged people. We considered that the elements of cognitive activity involved in reading picture books would be related to executive function. However, a previous study also found no intervention effect in older adults with high levels of global cognitive functioning (19). Therefore, we considered that middle-aged people with high levels of cognitive function may have also not been significantly affected.

As described above, the current results suggest that, even if an intervention with exactly the same content was performed at the same frequency during the same period of time, the effect of the intervention may differ depending on participants’ age and baseline cognitive function. In many intervention studies involving intellectual activity, the content of the intervention was limited only to participants with a specific level of cognitive function, and intervention studies have not been conducted with participants of different ages and levels of cognitive function (18). To develop intervention programs that are accessible to more people and to promote these programs more widely, it will be necessary to provide appropriate programs that suit the cognitive level and requirements of the target population.

The current study involved several limitations that should be considered. First, although we reviewed the causes of cognitive function changes according to the characteristics of the picture book reading program, we did not directly examine which elements of the program affected cognitive function. For example, it remains unclear whether verbal function was affected by the program content itself or by interacting with other participants. This is a potential limitation of examining programs that encourage behavior change in participants. Second, we used cognitive tests to evaluate the intervention effect. It is possible that the improvement in scores was a factor related to the test itself that made it easy to reflect the intervention effect. To control for the factor of the test itself, it will be necessary to select outcome measures for interventions that are sensitive and do not have substantial learning effects in middle-aged people. Furthermore, to clarify which elements of the program affect cognitive function in detail, it will be necessary to conduct more intervention studies that subdivide the elements of the program, and that combine brain imaging methods (e.g., functional magnetic resonance imaging) with cognitive testing. Finally, as a trend in hobbies, picture book reading is currently more popular among women, so there is a bias in the gender characteristics of the participants. Therefore, it should be noted that this result cannot be generalized to all people.

In the program examined in the current study, many participants joined volunteer groups after the intervention, volunteering to read picture books in their local communities, such as at nursery schools and elementary schools. The effects of volunteering have also been examined in older adults, and beneficial impacts have been reported in the suppression of hippocampal atrophy (39) as well as physical function and mental health (40, 41). Because the effects of this type of volunteering in middle-aged people have not previously been examined, long-term effects including volunteer activities after completing the program, and its effects on mental health and physical function should be investigated in future studies. Furthermore, this study was part of a project of the local government, and it was meaningful to find the effect of the intervention in an environment that was similar to the real world. The elements such as intellectual stimulation and social interaction included in this picture book reading program may be related to delaying future cognitive decline in middle-aged people. Most of the projects of the local governments related to countermeasures of dementia are mainly based on physical activity, but we consider it is also meaningful to adopt projects that focus on intellectual activities and social intellection.

In conclusion, the results indicated that our previous finding of an improvement in memory function in older adults was not found in middle-aged people. However, the findings suggest that the picture book reading program may affect lexical access ability in verbal function among middle-aged people. Because maintaining verbal function is important for daily communication, these findings suggest that this program may be a useful countermeasure for cognitive decline in middle-aged people.

## Data Availability Statement

The raw data supporting the conclusions of this article will be made available by the authors, without undue reservation.

## Ethics Statement

The studies involving human participants were reviewed and approved by the Institutional Review Board and Ethics Committee of the TMIG (Acceptance No. 0083). The patients/participants provided their written informed consent to participate in this study.

## Author Contributions

HS and YF: conceptualization and methodology. SO, DC, DY, KS, YK, and MK: data curation. AI, HS, and SO: formal analysis. AI, SO, DC, DY, KS, YK, MK, and HS: investigation. SO, TT, DC, KS, and HS: project administration. YF: supervision. SO, TT, and HS: visualization. AI and HS: writing–original draft. MK and YF: writing–review and editing. All authors contributed to the article and approved the submitted version.

## Conflict of Interest

The authors declare that the research was conducted in the absence of any commercial or financial relationships that could be construed as a potential conflict of interest.
