# Supplementary material for: Randomized Controlled Trial of the Picture Book Reading Program on Cognitive Function in Middle-Aged People
Source: Front Psychiatry. 2021 May 19;12:624487. doi: 10.3389/fpsyt.2021.624487 (PMC8170038; doi:10.3389/fpsyt.2021.624487)
Supplement: Supplementary file 1 [file Table_1.DOCX]

Supplementary A. Characteristics of participants enrolled but not in the final analysis (N=7).

|  | Age  (years) | Sex | Education  (years) | GDS-15  (Scores) | MMSE  (Scores) | Reason of dropped out |
| --- | --- | --- | --- | --- | --- | --- |
| IG |  |  |  |  |  |  |
| Participant A | 58 | female | ≧13 | 0 | 30 | Due to acute health problem. |
| Participant B | 60 | female | ≧13 | 4 | 29 | Refused to take the picture book classes. |
| Participant C | 63 | female | ≧13 | 7 | 28 | Due to the schedule conflict of the post test. |
| CG |  |  |  |  |  |  |
| Participant E | 64 | female | ≧13 | 3 | 30 | Due to the schedule conflict of the post test. |
| Participant F | 62 | female | ≧13 | 1 | 30 | Due to the schedule conflict of the post test. |
| Participant D | 64 | female | ≧13 | 3 | 29 | Due to the schedule conflict of the post test. |
| Participant G | 62 | female | ≦12 | 1 | 29 | Due to the schedule conflict of the post test. |

Abbreviations: N: Number of participants; IG: Intervention Group; CG: Active Control Group; GDS-15: 15- item Geriatric Depression Scale; MMSE: Mini-Mental State Examination.
